# Midwives’ experiences of formula supplementation for non-medical indications in the Swedish postnatal care context

**DOI:** 10.3389/fgwh.2026.1893484

**Published:** 2026-10-16

**Authors:** Pernilla Ny, Atika Khalaf, Tinde Dowler, Louise Karlsson, Li Thies-Lagergren

**Affiliations:** 1 Department of Health Sciences, Midwifery Research, Reproductive, Perinatal and Sexual Health, Faculty of Medicine, Lund University, Lund, Sweden; 2 The PRO-CARE Group, Faculty of Health Science, Kristianstad University, Kristianstad, Sweden; 3 Hind Bint Maktoum College of Nursing and Midwifery, Mohammed Bin Rashid University of Medicine and Health Sciences, Dubai Health, Dubai, United Arab Emirates; 4 Department of Obstetrics and Gynaecology, Skåne University Hospital, Malmö

**Keywords:** breastfeeding, formula supplementation, infant feeding, midwifery, postnatal care, qualitative research

## Abstract

**Background:**

Formula supplementation of breastfed infants for non-medical indications is discouraged in Swedish postnatal care. Still, supplementation rates remain high, with 20%-40% of infants receiving formula in postnatal wards. Early formula supplementation has been associated with reduced breastfeeding exclusivity and duration. Little attention has been given to the perspectives of healthcare professionals involved in supplementation decisions, and there is limited understanding of how midwives experience and navigate situations where formula is requested or considered for non-medical reasons.

**Aim:**

To explore midwives’ experiences of formula supplementation for non-medical indications in postnatal wards.

**Methods:**

This qualitative study used an inductive design with semi-structured interviews. Eleven midwives from three postnatal wards in Southern Sweden participated in individual interviews. The interviews were audio-recorded, transcribed verbatim analyzed using content analysis.

**Results:**

Three categories were identified: *Workplace culture is crucial, Negotiating infant feeding decisions* and *Moral and professional tensions.* Midwives described how workplace culture, guideline adherence, knowledge levels, and interprofessional dialogue influenced supplementation practices. Decisions about supplementation were often negotiated in interactions with parents, where midwives sought to facilitate informed decision-making while supporting breastfeeding. Supplementation for non-medical indications was frequently experienced as professionally challenging and emotionally difficult.

**Conclusion:**

Midwives’ experiences suggest that formula supplementation practices are shaped by organizational culture, professional alignment within the healthcare team, and interactions with parents. Strengthening breastfeeding-promoting postnatal care may therefore require organizational support, consistent education in breastfeeding support, and structures that enable midwives, who possess the strongest expertise, to actively participate in infant feeding decisions together with the mother.

## Introduction

Exclusive breastfeeding for the child's first six months of life and continued partial breastfeeding up to at least two years of age is recommended by the World Health Organization (1). Breastfeeding entails substantial short- and long-term health benefits for both mother and child. These include reduced maternal risk of breast- and ovarian cancer, cardiovascular disease, hypertension, and type 2 diabetes (2, 3), as well as improved maternal well-being and a lower risk of postpartum depression (4, 5). Breastfeeding provides infants with optimal nutrition and immune protection, lowering the risk of respiratory and gastrointestinal infections, otitis media, obesity, type 1 diabetes, sudden infant death syndrome (SIDS), and overall infant mortality (3).

Also, breastfeeding contributes to improved public health outcomes and environmental sustainability (3, 6–8). In Sweden, breastfeeding intention among pregnant women is high, with 95%–99% of first-time mothers reporting that they intend to breastfeed (46). Still, less than 72% of infants were exclusively breastfed during the first week of life (9).

Breastfeeding promotion in Sweden is guided by national policies such as the Baby-Friendly Hospital Initiative (BFHI) and the *Ten Steps to Successful Breastfeeding* (10). Evidence-based professional support is crucial for establishing breastfeeding and promoting positive maternal experiences, whereas inconsistent guidance can undermine maternal confidence (11, 12). Within Swedish maternity care, midwives hold primary responsibility for breastfeeding support, including assessment, evidence-based counselling, and promoting maternal autonomy alongside infant safety (13). Due to midwifery shortages in Sweden, several regions have replaced midwives with registered nurses on postpartum wards (10). Postpartum care quality relies on continuity, professional competence, and consistent guidance, factors mothers identify as essential to their postnatal care experience (14, 15). These conditions place midwives in a challenging position, balancing evidence-based breastfeeding support with organizational constraints.

Postnatal hospital stays in Sweden are short (10), leaving limited time for adequate breastfeeding support. Despite the aims of the BFHI (16), gaps in breastfeeding competence among healthcare professionals persist (17). Women also report feeling forced to introduce formula despite intending to breastfeed (18).

Infant formula was introduced in Sweden in the 1940s and is currently regulated under European Union legislation to ensure nutritional adequacy (Eur-Lex Regulation 609/2013). Concerns of how formula may affect breastfeeding, the WHO introduced the International Code of Marketing of Breastmilk Substitutes in 1981 to safeguard and promote breastfeeding (19). Formula supplementation, medically indicated in cases like prematurity, hypoglycemia, hyperbilirubinemia or abnormal growth (20) disrupts breastfeeding establishment and reduces exclusivity and duration (20–23).

Despite indisputable recommendations and advice against non-medical use of formula, the global formula market has continued to expand, and formula provided to healthy, full-term breastfed infants remains widespread in Swedish postnatal care, with national rates averaging 30% (7, 24). Supplementation frequently occurs in the absence of medical indications, due to maternal request, perceived infant dissatisfaction or crying, limited breastfeeding knowledge, and organizational constraints such as time pressure in clinical practice (25, 26).

Little attention has been given to the perspectives of healthcare professionals involved in supplementation decisions, and there is limited understanding of how midwives in Sweden experience and navigate situations where formula is requested or considered for non-medical reasons. Understanding midwives’ experiences may provide important insights into the clinical and organizational factors shaping supplementation practices and inform strategies to strengthen breastfeeding-promoting postnatal care. Therefore, the aim of this study was to explore midwives’ experiences of formula supplementation for non-medical indications in Swedish postnatal wards.

## Materials and methods

### Design

This study used a qualitative descriptive design, with semi-structured individual interviews. An inductive analytic approach was adopted, allowing patterns and categories to be derived from the data (27, 28).

Participants were recruited from three postnatal wards within a single Swedish region in southern Sweden. Participants (eleven) were licensed midwives working in postnatal care, together with other professions, including nurses. Inclusion criteria were registered midwives with current or recent experience in postnatal wards and of non-medically indicated formula supplementation in healthy, full-term infants. No exclusion criteria were applied.

A convenience sampling strategy was used to recruit information-rich participants with direct experience relevant to the study aim (27). Initial recruitment took place through ward managers at one of the postnatal wards, who were asked to distribute written study information to eligible midwives. When recruitment was limited, additional invitations were shared to other postnatal wards (two) through digital professional communication channels and workplace group chats used by midwives (27).

Two members of the research team (TD and LK) conducted all the interviews, with prior experience as specialist pediatric nurses and clinical experience in postnatal care settings, including experience of breastfeeding support and formula supplementation. PN, AK, and LTL are familiar with supplementation on medical indications and with breastfeeding-promoting care.

Semi-structured interviewing was chosen to ensure coverage of key topics while allowing participants to describe their experiences in their own terms (27). One pilot interview was conducted. The pilot interview was used to test the interview guide and refine the interviewing technique. Following this, one additional question was added to improve alignment with the study aim. The pilot interview was not included in the analysis.

In accordance with Swedish legislation, this study does not require approval from the Swedish Ethical Review Authority as it does not involve patients or sensitive data. All procedures were carried out in compliance with the ethical standards established by the Committee on Publication Ethics (COPE). It is the authors’ responsibility to protect the informants from any harm. The authors adhered to the ethical principles, including the requirements of information, confidentiality and utilization (29).

Interviews were conducted between November 2024 and April 2025. Participants chose the time and mode of the interview. Three interviews were conducted face-to-face, and eight via Zoom. Interviews lasted between 18 and 49 min (mean: 30 min). Participants received written information about the study purpose, procedures, confidentiality, voluntary participation, and their right to withdraw at any time without consequences, according to the Swedish Ethical Review Act (29) and the Declaration of Helsinki (30).

Written informed consent was obtained before participation. In online interviews where written signing was not practicable, verbal consent was audio-recorded at the start of the interview, after confirming that the participant had read and understood the study information.

At the start of each interview, participants were invited to describe their educational background and clinical experience before being asked to reflect on their experiences of formula supplementation in postnatal care.

The first six interviews were conducted with both researchers present to establish a consistent interviewing approach. In these interviews, one researcher led the conversation while the other observed, managed technical aspects, and asked occasional follow-up questions where appropriate. The remaining interviews were then conducted individually by the two researchers.

All interviews were audio-recorded with participants’ consent and transcribed verbatim.

Recruitment and data collection proceeded concurrently. During the later interviews, the researchers observed increasing repetition of the data and fewer new insights relevant to the study aim. Recruitment was therefore discontinued when the research team assessed that the dataset provided sufficient depth, richness, and variation to address the study aim and support robust category development.

### Data analysis

The data were analyzed using qualitative content analysis according to Graneheim et al. (31) suitable for identifying patterns, similarities, and variations in participants’ experiences. Analysis began with repeated reading of the verbatim transcripts to obtain an overall sense of the material. Interviews conducted by one researcher were transcribed by the other researcher, which facilitated familiarity with the full dataset. Meaning units relevant to the study aim were then identified in the text. Where a meaning unit contained more than one central idea, it was divided into separate units. The meaning units were condensed while preserving their core content and then labelled with codes describing their manifest meaning (31).

All the researchers compared codes for similarities and differences and grouped related codes into preliminary subcategories. These subcategories were reviewed and refined through iterative discussion until all coded data could be meaningfully organized. Broader categories were then developed by abstracting across subcategories and describing the underlying patterns in the data at a higher interpretive level (31). Throughout the analytic process, the researchers moved back and forth between the original transcripts, condensed meaning units, codes, and emerging categories to ensure internal consistency and grounding in the data. An example of the analytic process is presented in Table 1.

**Table 1:** Example of the qualitative content analysis process.

Meaning unit (quotation)	Condensed meaning unit	Code	Subcategory
“A so-called ‘rough start’, even though it wasn't really a rough start. Possibly a large baby, even though our guideline states that we can wait before supplementing them” (Participant 11)	Infant supplemented due to perceived “rough start” or large size despite guidelines indicating supplementation can wait	Supplementation despite guidelines indicating waiting is appropriate	Inconsistent adherence to guidelines
“It still becomes this kind of random giving of formula, with no real structure, and no specific reason” (Participant 8)	Formula given in an unstructured way without a clear clinical indication	Supplementation without clear indication	

### Ethical considerations

Because the interviews addressed practices that may not always align with guidelines, there was a potential risk of discomfort or concern about professional judgment. To minimize this risk, participants were informed that the purpose was to explore experiences of practice rather than to evaluate individual performance. Confidentiality was emphasized throughout.

Audio files and transcripts were stored on password-protected devices and in secure software environments without external synchronization. Recordings were pseudonymized, and the key linking codes to identities was stored separately. Care was taken in reporting the findings to ensure that individual participants and workplaces could not be identified.

## Results

Altogether, a total of eleven midwives participated in the study. Their professional experience ranged from one to 35 years (median: three years). Approximately half of the participants had undertaken advanced-level breastfeeding education, while all had completed workplace-based breastfeeding training. Some also had specialist training as lactation consultants (IBCLC). In Sweden, breastfeeding and lactation management are part of midwifery education, with opportunities for further specialization through continuing education, resulting in varied levels of formal lactation training among participants.

The findings reflect the experiences of eleven midwives regarding formula supplementation for non-medical indications in postnatal wards. Participants described not only their own clinical practices but also observations of colleagues’ practices and the broader workplace culture surrounding supplementation.

The analysis yielded three main categories and eight subcategories (Table 2).

**Table 2:** Categories and subcategories.

Category	Subcategories
Workplace culture is crucial	• Inconsistent adherence to guidelines • Knowledge and awareness influence clinical decision-making • The role of professional dialogue
Negotiating infant feeding decisions	• Facilitating informed decision-making • Shaping parents’ future feeding practices
Moral and professional tensions	• Resisting non-medically indicated supplementation • Supporting maternal capacity for breastfeeding • Emotional responses to supplementation decisions

### Workplace culture is crucial

Midwives described how workplace culture, rather than shared professional perspectives, strongly influenced how supplementation was approached in practice. Postnatal wards where health care professionals, such as midwives, nurses, physicians, and assistant nurses, shared a common understanding of breastfeeding-supportive care were perceived to have lower rates of non-medically indicated supplementation. Conversely, when such consensus was lacking, supplementation was more frequently initiated outside established guidelines.

Internal training and ongoing professional discussions were described as important mechanisms for developing a shared approach to infant feeding practices. Where such structures were present, midwives perceived greater consistency in clinical decision-making and a more restrictive approach to supplementation.

#### Inconsistent adherence to guidelines

Participants described situations in which clinical guidelines regarding supplementation were not consistently followed. One commonly mentioned issue was incomplete documentation. Supplementation sometimes occurred without the indication or the intervention itself being recorded in the infant's medical record.

“Sometimes it is documented that supplementation was given… but if it was used, for example, just to encourage the baby during breastfeeding… then it isn't always written down.” (Participant 9)

Participants also described instances in which supplementation was initiated based on informal routines or assumptions rather than on documented clinical assessment. For example, infants perceived as unsettled, jittery, or breathing rapidly were sometimes supplemented without further evaluation or consultation with a physician.

“If we suspect low blood sugar, we should check it… but we shouldn't give supplementation just because the baby is a bit shaky.” (Participant 11)

Midwives also described how supplementation could occur almost automatically in certain situations or without a clear rationale. Organizational factors such as workload, time pressure, and variable levels of health care professionals’ interest in breastfeeding support were perceived as contributing to this practice.

“Sometimes supplementation happens and no one can really explain why… it may simply be due to stress, lack of time, or, unfortunately, sometimes lack of interest.” (Participant 5)

#### Knowledge and awareness influence clinical decision-making

Participants emphasized that knowledge about breastfeeding and supplementation played a central role in shaping clinical practice. Postnatal wards that regularly provided internal training on breastfeeding were perceived to have higher healthcare professionals’ awareness and more consistent adherence to guidelines.

Such training fostered a shared understanding among midwives and assistant nurses, leading to more reflective decision-making and greater caution in initiating supplementation.

“You start thinking more carefully… you don't just supplement automatically. The internal training has really contributed to that.” (Participant 8)

In contrast, participants working in environments without structured breastfeeding education described more variability in practice. In these settings, supplementation could sometimes be initiated by other healthcare professionals without the responsible midwife's consultation.

“Sometimes assistant nurses just give formula quite freely… and that really comes down to lack of knowledge.” (Participant 1)

#### The role of professional dialogue

Participants emphasized the importance of interprofessional communication in decision-making about supplementation. Midwives considered themselves central to these decisions because breastfeeding support is part of their professional scope of practice.

When colleagues engaged in dialogue, whether midwives, assistant nurses, or physicians, it often led to more considered decision-making. In some cases, such discussions led to supplementation being avoided.

“There can be a culture where people simply run off to get formula without asking or discussing it first.” (Participant 10)

Participants also described how professional experience influenced their willingness to question physicians’ decisions about supplementation.

“You need to feel secure enough to question it… a newly graduated nurse will probably just give the formula.” (Participant 1)

### Negotiating infant feeding decisions

Midwives described how their interactions with parents played a central role in decisions about supplementation. Their approach involved balancing parental wishes, providing evidence-based information, and supporting breastfeeding where possible.

#### Facilitating informed decision-making

Participants highlighted the importance of ensuring that parents receive sufficient information to make informed decisions about supplementation. When parents requested formula, midwives often sought to explain how supplementation could affect breastfeeding outcomes.

“I always try to explain what it means if we introduce formula and what the possible consequences might be later.” (Participant 10)

Midwives described asking parents about their reasons for requesting supplementation in order to tailor information and support.

“You usually get further by being curious, asking why the parents want supplementation and what experiences they have.” (Participant 4)

However, providing information could be challenging when parents lacked prior knowledge about breastfeeding or had unrealistic expectations about infant behavior, such as expecting newborns to sleep for extended periods. Fatigue and emotional vulnerability following childbirth could also affect parents’ ability to process information.

#### Shaping parents’ future feeding practices

Participants emphasized their responsibility to guide parents and support their confidence in caring for their infant without relying on supplementation. Midwives perceived their actions during the hospital stay as shaping parents’ future feeding practices.

“What we do on the ward can set the tone for what parents continue doing at home.” (Participant 9)

Participants described situations in which supplementation appeared to reassure parents in the short term, particularly when an unsettled infant appeared calmer after receiving formula. However, this could increase the likelihood that parents would request supplementation again.

Midwives, therefore, underlined the importance of reflecting on their actions and providing guidance that strengthened parental confidence in breastfeeding and infant care.

### Moral and professional tensions

Participants described supplementation for non-medical indications as emotionally and professionally challenging. These practices were perceived as conflicting with their professional responsibility to promote breastfeeding.

#### Resisting non-medically indicated supplementation

Midwives described actively attempting to prevent supplementation when no medical indication was present. This stance was grounded in their knowledge of breastfeeding physiology and the potential impact of supplementation on breastfeeding establishment.

“Supplementation without medical indication is something we constantly have to work against, because it happens all the time.” (Participant 11)

Participants explained that maintaining a restrictive approach required ongoing effort, particularly in organizational environments where supplementation could otherwise occur easily.

#### Supporting maternal capacity for breastfeeding

Participants also highlighted the importance of recognising the resources already present in the mother–infant dyad. Instead of initiating supplementation, midwives described supporting mothers in expressing colostrum or adjusting breastfeeding techniques.

“Instead of giving formula, we now try more often to help the mother express milk or hand-express colostrum.” (Participant 8)

Midwives suggested that supplementation could sometimes be avoided by conducting a more comprehensive assessment of the breastfeeding situation and developing an individualized plan.

#### Emotional responses to supplementation decisions

Participants described experiencing frustration, sadness, and professional discomfort when supplementation occurred without medical indication. Awareness of the potential impact on breastfeeding outcomes contributed to these feelings.

“Initially, I found it quite difficult to give formula… it felt wrong somehow, like the last thing I wanted to do.” (Participant 7)

These emotional responses reflected a broader perception among participants that supplementation for non-medical reasons represented a departure from the core values of midwifery practice.

## Discussion

This present study is, to the best of our knowledge, the first to address midwives’ experiences regarding formula feeding from a qualitative perspective. The findings suggest that non-medically indicated formula supplementation cannot be understood solely as an individual clinical decision. Rather, it appears to be produced at the intersection of organizational culture, professional competence, interprofessional communication, and relational care in encounters with families. Breastfeeding must be understood in the context of women's and children's rights, as well as within the framework of sustainable development.

According to the Right of the Child Act (32) every child has the right to good health, while women have the right to make informed choices about their health in line with Sustainable Development Goal 3 on ensuring healthy lives and promoting well-being for all at all ages (33). This also aligns with human-rights-based commitments to gender equality, including the Swedish six sub-goals on gender equality promoted by the (34), which affirms women's right to give and receive care on equal terms.

### Organizational drivers of supplementation practices

Supplementation practices were influenced by the extent to which the clinical team shared a common breastfeeding-supportive approach. Where such alignment existed, supplementation was perceived as less frequent and more clinically justified. In contrast, where it was lacking, practices were described as inconsistent and supplementation became normalized. These finding aligns with previous research showing that health care professionals’ knowledge, attitudes, and consistency of messaging are important determinants of infant feeding practices in postnatal care (35–38).

Uneven breastfeeding knowledge among postpartum healthcare professionals contributes to inconsistent breastfeeding support and advice (37), while lack of team coherence may undermine collaborative care (35). The present findings extend this literature by suggesting that organizational misalignment may also influence how supplementation is initiated, discussed, documented, and challenged.

Participants’ accounts of incomplete documentation and supplementation based on informal rather than clinical grounds are noteworthy. Similar patterns have been reported elsewhere (37). The findings suggest that non-medically indicated supplementation may become normalized in routine practice, despite its implications for clinical accountability and interprofessional communication.

The midwives also linked non-indicated supplementation to workload, time constraints, and variable interest in breastfeeding support. This aligns with previous research identifying organizational pressures as barriers to effective breastfeeding support in postnatal care (39–41). Our findings suggest that supplementation were sometimes used as a pragmatic response to clinical strain rather than infant need, which is concerning given the Baby Friendly Hospital Initiative's emphasis on organizational responsibility for providing conditions that support breastfeeding-promoting care (42).

Viewed through a midwifery lens, these findings point to an important structural issue: even where midwives possess the necessary competence, organizational conditions may limit their ability to practice in accordance with evidence and professional standards. This interpretation is consistent with Halldorsdottir and Karlsdottir (43) conceptualization of professional midwifery as integrating competence, judgement, caring, and interpersonal skill within a supportive practice environment.

### Infant feeding decisions in parent-midwife interactions

The second major finding concerned the relational work involved in supplementation decisions. Midwives described their role as facilitating informed decision-making through dialogue, tailored information, and support. Participants highlighted the importance of exploring why parents requested supplementation, addressing expectations about newborn behavior, and communicating the possible implications of supplementation for breastfeeding.

These finding aligns with Swedish and international guidance emphasizing that parents should receive clear, evidence-based support to enable informed infant feeding decisions (1, 13, 42). It also reflects previous research showing that women value individualized, realistic, and responsive breastfeeding support (35, 44, 45). In the present study, midwives described informed choice as an ethically grounded process that includes ensuring parents understand infant behavior and the potential consequences of formula supplementation.

Importantly, participants described parents’ requests for supplementation as often occurring in the context of fatigue, uncertainty, limited breastfeeding knowledge, and the emotional intensity of the early postnatal period. These circumstances may affect parents’ ability to fully process information, highlighting the importance of skilled, responsive communication. Consistent with Chesnel et al. (35), the findings underscore the need for healthcare professionals to carefully manage the inherent power imbalance in conversations about breastfeeding and supplementation.

Participants also described ward-based supplementation as influencing feeding practices after discharge. When formula appeared to calm an unsettled infant, parents often perceived it as an effective solution and were more likely to repeat it at home. This finding is clinically significant, as it suggests that early institutional practices may shape parental confidence and feeding decisions. It also supports evidence linking early supplementation to reduced breastfeeding duration and exclusivity (21, 23), while highlighting how supplementation may acquire symbolic meaning as a successful response to uncertainty. From a midwifery perspective, supporting parents in understanding normal newborn behavior, tolerating uncertainty, and recognizing maternal capacity may be as important as technical breastfeeding support. This underscores the importance of relational continuity and confidence-building care (43).

### Formula as risk, reassurance, and moral tension

The third major finding was that non-medically indicated supplementation was experienced by many midwives as emotionally difficult and professionally dissonant. Participants described frustration, sadness, and a sense of failure when supplementation occurred without clear clinical indication. They also described actively resisting such supplementation and seeking alternatives that supported maternal breastfeeding capacity.

This finding suggests that supplementation is not experienced merely as a neutral feeding intervention, but as a practice loaded with professional and moral meaning. Because breastfeeding support is explicitly included in the Swedish midwifery scope of practice (13), supplementation without medical indication may be perceived as conflicting with the core midwifery responsibility to protect, promote, and support breastfeeding. The emotional responses reported by participants may therefore reflect moral tension between what midwives believe constitutes good care and what occurs in practice, under organizational, relational, or cultural pressure, as performed by midwives and nurses.

The findings suggest that midwives’ resistance to supplementation was often grounded in a commitment to preserving breastfeeding by identifying and mobilizing resources within the mother-infant dyad, such as expressing colostrum, optimizing breastfeeding technique, or developing individualized feeding plans. Rather than opposing parental wishes, this approach reflected an effort to support breastfeeding while addressing the family's needs. This is consistent with evidence that women benefit from individualized support that strengthens confidence in their breastfeeding capacity (44, 45). At the same time, participants’ emotional burden should not be overlooked. When midwives repeatedly encounter practices they perceive as inconsistent with evidence or professional values, this may generate frustration and moral residue. Although this study did not specifically examine moral distress, the findings suggest that future research could usefully explore whether repeated exposure to non-indicated supplementation contributes to professional dissatisfaction or ethical strain in postnatal care.

### Strengths and limitations

A qualitative design with semi-structured interviews was appropriate for exploring midwives’ experiences of a complex clinical practice (27). Trustworthiness was addressed through credibility, dependability, confirmability, and transferability. Credibility was supported by participants’ direct experience, open-ended questions, and close engagement with the data (27). Dependability was strengthened by an interview guide, researcher discussions, and transparent analysis (27, 28). Confirmability was enhanced by reflexive discussions of pre-understanding and repeated checks of interpretations against the transcripts (27).

Transferability may be limited as data were collected from three postnatal wards within a single Swedish region. However, the region accounts for a substantial proportion of births in Sweden and was therefore considered to provide sufficient variation in experiences relevant to the study aim and the wide variation in participants’ professional experience may support the applicability of the findings to similar contexts (27).

Limitations include convenience sampling and snowball sampling, which may have attracted participants with a strong interest in breastfeeding, and the fact that some interviews were conducted at workplaces, which could have influenced disclosure. In addition, the findings reflect only midwives’ perspectives, as exploring the views of other professional groups was beyond the scope of the study. However, allowing participants to choose location and mode may have increased their sense of safety and control (27). Joint and individual interviews may have introduced slight variation in style, but could also enrich the data (31).

Participants described their own and colleagues’ practices and the local culture around supplementation, treated as valid expressions of midwives’ experiences in postnatal care. The interviews were conducted by LK and TD, at the time specialist nurses in pediatrics, with reliability strengthened by a senior researcher, all registered midwives, experienced in qualitative analysis. All authors read, analyzed, and discussed the findings, and the team had prior experience in postnatal breastfeeding care and formula provision.

### Implications for practice, education, and policy

The findings have several implications for postnatal care. First, reducing non-medically indicated supplementation requires not only individual professional commitment but also organizational support for coherence and a clearly defined leading role for the midwife in breastfeeding support. Regular interprofessional training, clear local protocols, and structured team dialogue are key to delivering consistent, breastfeeding-promoting care.

Second, the findings underline the need to protect midwives’ professional responsibility in infant feeding decisions. Organizations must ensure midwives have the authority, time, and support to lead breastfeeding-promoting care and challenge non-indicated supplementation. Breastfeeding support should be anchored in midwifery practice, with timely, realistic information about newborn behavior, breastfeeding physiology, and the consequences of supplementation, preferably offered during pregnancy. This is particularly critical during short postnatal stays, where early experiences can shape subsequent feeding practices at home.

## Conclusion

Non-medically indicated formula supplementation was experienced by midwives as shaped by organizational culture, interprofessional alignment, and communication with parents. Restrictive and reflective practices were supported where there was a shared understanding and dialogue, while time pressure and fragmented knowledge contributed to the normalization of formula supplementation outside medical indications. The findings indicate that breastfeeding-promoting postnatal care cannot rely solely on midwives’ individual competence but requires organizational conditions that support evidence-based, ethical, and women-centred care, in line with the Sustainable Development Goals.

## Data Availability

The datasets presented in this article are not readily available because transcribed individual data can not be shared. Requests to access the datasets should be directed to pernilla.ny@med.lu.se.
